# Teaching Medical Students How to Ask Older Adults What Matters Using Simulated Patients

**DOI:** 10.1093/geroni/igaa057.1940

**Published:** 2020-12-16

**Authors:** Laurin Mack, Jamie Cvengros, Erin Emery-Tiburcio

**Affiliations:** Rush University Medical Center, Chicago, Illinois, United States

## Abstract

It is vital the workforce is prepared to meet the medical needs of our aging population. Asking older adults What Matters is an important aspect of excellence in clinical care. During a small group session in a two-year communication skills course, second year medical students (N=149) at Rush were taught how to ask What Matters as part of the 4Ms. Students then completed a video recorded Communication Skills Lab with a simulated older adult patient as they practiced how to discuss What Matters. Students then met with their instructors in individual feedback sessions to review the video and discuss strengths and areas for improvement in communicating with older adults. Students then completed a Clinical Skills Assessment for formal testing of their communication skills with older adults. Outcomes of the summative assessment will be presented and recommendations for integrating 4Ms into existing medical school and allied health curriculum will be discussed.

